# High-fidelity simulation self-training enables novice bronchoscopists to acquire basic bronchoscopy skills comparable to their moderately and highly experienced counterparts

**DOI:** 10.1186/s12909-018-1304-1

**Published:** 2018-08-07

**Authors:** Martin Veaudor, Laurence Gérinière, Pierre-Jean Souquet, Loïc Druette, Xavier Martin, Jean-Michel Vergnon, Sébastien Couraud

**Affiliations:** 1 Service de Pneumologie Aigue Spécialisée et Cancérologie Thoracique, Institut de Cancérologie des Hospices Civils de Lyon, Centre Hospitalier Lyon Sud, 69310 Pierre Bénite, France; 2 Stratégie d’Apprentissage des Métiers de Santé en Environnement Immersif, Université Lyon 1, 69008 Lyon, France; 3 Service de Pneumologie et oncologie thoracique, CHU St Etienne, Hôpital Nord, 42270 Saint-Priest en Jarez, France; 4 EMR 3738 Ciblage thérapeutique en oncologie, Faculté de médecine Lyon Sud Charles Mérieux, Université Lyon 1, 69600 Oullins, France

**Keywords:** High-fidelity simulation, Flexible bronchoscopy, Medical education, Self-training

## Abstract

**Background:**

We sought to determine whether a self-training program on a high-fidelity flexible bronchoscopy (FB) simulator would allow residents who were novices in bronchoscopy to acquire competencies similar to those of experienced bronchoscopists as concerns the visualization of the bronchial tree and the identification of its anatomical elements.

**Methods:**

We performed a prospective cohort study, categorizing bronchoscopists into three groups according to their experience level: novice (Group A, no FBs performed, *n* = 8), moderate (Group B, 30 ≤ FBs performed ≤200, *n* = 17) or high (Group C, > 200 FBs performed, *n* = 9). All were initially evaluated on their ability to perform on a high-fidelity FB simulator a complete visualization/identification of the bronchial tree in the least amount of time possible. The residents in Group A then completed a simulation-based self-training program and underwent a final evaluation thereafter.

**Results:**

The median total procedure time for Group A fell from 561 s (IQR = 134) in the initial evaluation to 216 s (IQR = 257) in the final evaluation (*P* = 0.002). The visualization and identification scores for Group A also improved significantly in the final evaluation. Resultantly, the overall performance score for Group A climbed from 5.9% (IQR = 5.1) before self-training to 25.5% (IQR = 26.3) after (*P* = 0.002), thus becoming comparable to the overall performance scores of Group B (25.3%, IQR = 13.8) and Group C (22.2%, IQR = 5.5).

**Conclusions:**

Novice bronchoscopists who self-train on a high-fidelity simulator acquire basic competencies similar to those of moderately or even highly experienced bronchoscopists. High-fidelity simulation should be rapidly integrated within the learning curriculum and replace traditional, in-patient learning methods.

**Electronic supplementary material:**

The online version of this article (10.1186/s12909-018-1304-1) contains supplementary material, which is available to authorized users.

## Background

Flexible bronchoscopy (FB) is a safe procedure, with rates of 0.05 and 0.5% for attributable mortality and major complications respectively [1–3]. Although FB has a low rate of minor complications [1], the tolerability of the procedure for the patient is mediocre [4], especially for prolonged procedures [5]. Considering procedure tolerability, many patients report anxiety for FB, but may be reassured by the bronchoscopist’s experience [4]. In contrast, the risk of complications may be significantly higher when FB is performed by an inexperienced bronchoscopist [6].

Simulation-based FB training enables learners to hone their skills in a safe training environment, and become at ease with the procedure before moving on to patients. Thus, simulation training should be able to contribute improving the tolerability and safety of FB procedures performed by new practitioners. Several methods of bronchoscopy simulation have been developed and studied. These include animal models [7–9], low-fidelity trainers [10–15], and, since the early 2000s, high-fidelity virtual-reality simulators [16–20]. In a meta-analysis published in 2013, bronchoscopy simulation provided clear benefits as concerned practitioner skills and behaviors and moderate benefits as concerned procedure length [21]. However, many of the studies included in that meta-analysis were performed in the settings of otolaryngology or anesthesiology. Bronchoscopy in those fields is relatively simple; it does not involve detailed visualization of the bronchial tree as is the case in pulmonology.

In 2015, a panel of American experts published propositions for the development of lung bronchoscopy training programs [22]. They suggested that high-fidelity simulation should be available to all learners. Despite those recommendations, FB training continues to be largely based on the supervised “see one, do one, teach one” model [23]. Today however, it seems difficult to justify the subjection of patients to examinations performed by learners who are still in the cognitive or integrative phase of their training when there are simulators developed specifically for FB [24].

For the work presented here, we sought to determine whether a high-fidelity FB simulation self-training program would allow novice bronchoscopists to acquire competencies similar to those of trained bronchoscopists as concerns the visualization of the bronchial tree and the identification of its anatomical elements.

## Methods

### Study type and objectives

The primary objective of this prospective cohort study, carried out between October 2015 and September 2016, was to assess FB skill acquisition in novice bronchoscopists who self-trained on a high-fidelity bronchoscopy simulator (Simbionix Bronch Mentor), and compare their skills to those of moderately and highly experienced bronchoscopists (Additional file 1: Table S1). The capacity of this simulator to discriminate the competency (dexterity) and anatomical knowledge of trainees as a function of their past experience was demonstrated in a 2014 validation study [25]*.*

The secondary objectives were to determine the ability of the simulator to discriminate the skill levels of bronchoscopists as a function of their past experience and to evaluate student/user satisfaction with the simulator.

### Study population

All residents and pulmonologists included in the study practiced in the Auvergne Rhône-Alpes region of France. Participants were categorized according to their experience in bronchoscopy as commonly classified in the literature:

- Group A (novices, no experience): This group comprised first semester pulmonology residents. Eligibility criteria for this cohort were no former experience with FB (in patients or in simulation) and commitment to completing the entire training program and undergoing the initial and final evaluations.
- Group B (moderate experience): This group comprised residents and recently licensed pulmonologists who had themselves performed at least 30 FBs but less than 200 FBs in patients. Indeed, Wahidi et al. showed that pulmonary fellows learning of FB displayed a step upward curve in the 30 first bronchoscopies [18]. None had never received high-fidelity simulator training.
- Group C (extensive experience): This group comprised pulmonologists who had performed more than 200 FBs during their career - a commonly admitted threshold [16, 26]. Again, none had never received high-fidelity simulator training.

Groups A and B were composed largely of residents seeking certification in pulmonology and following the Harmonising Education in Respiratory Medicine for European Specialists (HERMES) program of the European Respiratory Society (ERS). HERMES comprises a specific module for bronchoscopy [27].

### Study organization

**Fig. 1:**
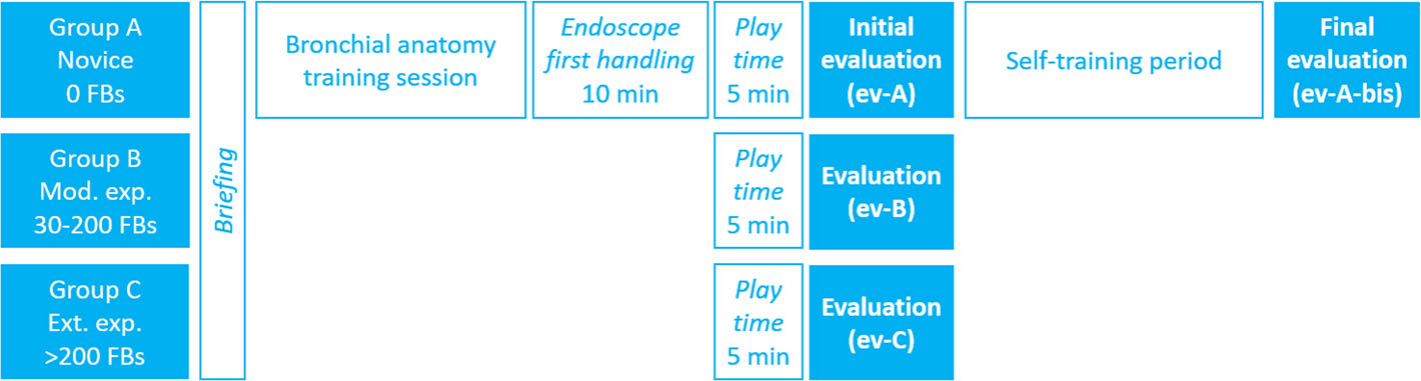
Study design

All three groups received an initial briefing to present the simulator and its operation. Group A benefited additionally from a short presentation on endobronchial anatomy and a special play-time dedicated to their first handle of an endoscope (10 min). Thereafter, all groups were given a 5 min “play time” to familiarize themselves with the simulator (Fig. 1).

Then, all of the groups were subjected to an initial evaluation (ev-A, ev-B and ev-C). Groups B and C underwent no further evaluations. Subsequently, the members of Group A were invited to use the simulator autonomously for as long as they wished, over one or several sessions (self-training). When they felt ready, the members of Group A requested a new evaluation (ev-A-bis) with the evaluator. Ev-A-bis was strictly identical to ev-A.

### Performance evaluation

The ability of each participant to perform a complete visualization of the bronchial tree, up to segmental bronchi, in as little time as possible was evaluated using the “Essential Bronchoscopy” module programmed within the simulator. The participants were required to visualize all anatomical structures and identifying them orally. They could use common anatomical names or Boyden classifications [28] according to their personal preferences. The exercise included the passage through the vocal cords. The evaluator noted participant performance on a dedicated form:

- The anatomical structures visualized during the procedure were marked each with a 1 and summed to establish a visualization (V) score from 0 to 28 points.
- The structures correctly identified were marked each with 1 and summed to establish an identification (I) score from 0 to 28 points.
- The sum of the two preceding scores provided the total (T) score (T = V + I), which ranged from 0 to 56.

At the end of the exercise, the evaluator noted the other performance variables provided by the simulator:

- Total procedure time, which was set at a maximum of 15 min;
- Percentage of time with scope-wall contact and the percentage of time with the scope at mid-lumen, both used to evaluate endoscope dexterity.

An overall performance (P) score, similar to that used in the seminal study by Ost et al. [17], was defined as the percentage of the number of segments correctly visualized and identified divided by the procedure time.

$$ P\left(\%\right)=\frac{V+I}{Time}\times 100 $$

The evaluations were conducted by two chest physicians who mutually developed a process to ensure objective and reproducible assessments.

### Satisfaction survey

After their evaluations, the participants were asked to respond to a questionnaire assessing their satisfaction with the simulator. The eight assessed items were rated from 0 (ineffective) to 6 (excellent) and summed to determine a global satisfaction score ranging from 0 to 48.

### Ethics considerations

The study was approved by the local ethic committee of Lyon University hospital on 22/09/2015. Signed consent was obtained from all participants before the first evaluation in line with French law (non-interventional study). Data collected were strictly anonymous.

### Statistical analyses

Categorical variables were expressed as percentages and continuous variables as medians and interquartile ranges (IQR). The Kruskal-Wallis test was used to compare distributions of continuous variables across more than two groups and the Mann-Whitney U test for comparisons between two groups. Statistical significance was set at *p* < 0.05. Statistical analyses were performed using SPSS version 19.0 (IBM Corp. Armonk, NY, USA).

## Results

### Participants

Thirty-four participants were enrolled during the study period, eight in Group A (novice) 17 in Group B (moderately experienced) and nine in Group C (highly experienced). Their characteristics are summarized in Table 1. We initially invited 22 novice bronchoscopists (Group A) to participate the initial evaluation; but only eight completed the full self-training sessions and the final evaluation. Group A was thus limited to those eight participants. We found no significant difference in the overall performance score between participants who dropped-out the program and those who complete it (7.99 [IQR1.9] vs. 5.93 [IQR 5.1]; *p* = 0.290). During the self-training period, seven of the Group A participants performed 20 or more FB simulations whereas one performed only 5.

**Table 1:** Participant characteristics for Groups A, B and C

	Group A	Group B	Group C
	No experience (*n* = 8)	Moderate experience (*n* = 17)	Extensive experience (*n* = 9)
Sex			
Female	3 (38%)	8 (47%)	5 (56%)
Male	5 (62%)	9 (53%)	4 (44%)
Learning hospital affiliation			
Lyon	7 (88%)	14 (82%)	9 (100%)
Clermont Ferrand	1 (12%)	1 (6%)	0
Grenoble	0	2 (12%)	0
Title			
MD thesis	0	2 (12%)	9 (100%)
Resident	8 (100%)	15 (88%)	0
Number of validated semesters (residents)			
0–2	4 (50%)	3 (20%)	–
3–5	2 (25%)	7 (41%)	–
6–8	2 (25%)	5 (29%)	–
Experience since thesis (licensed physicians)			
0–5 years	–	2 (100%)	2 (22%)
> 5 years	–	0	7 (78%)

### Initial evaluations

No significant differences were observed between the initial evaluations of Group B (ev-B) and Group C (ev-C) (Table 2). The median overall performance scores for ev-B and ev-C were respectively 25.3% (IQR = 13.8) and 22.2% (IQR = 5.5) (*p* = 0.5). There were no significant differences among the other ev-B and ev-C variables, (visualization score, identification score, scope-wall contact time, scope at mid-lumen time).

**Table 2:** Comparison of performances of Groups A, B and C in the initial evaluation

	Group A	Group B	Group C	*p* value (B vs C)	*p* value (A vs B vs C)
	No experience (*n* = 8)	Moderate experience (*n* = 17)	Extensive experience (*n* = 9)		
Visualization score (median [IQR])	25 [6]	27 [1]	28 [3]	0.545	0.147
Identification score (median [IQR])	8 [11]	24 [8]	20[9]	0.144	0.001
Total procedure time in seconds (median [IQR])	561 [134]	198 [112]	210 [77]	0.872	< 10^−4^
Overall performance score (%) (median [IQR])	5.9 [5.1]	25.3 [13.8]	22.2 [5.5]	0.5	< 10^−4^
Scope-wall contact time (%) (median [IQR])	60.5 [18]	62 [8]	63 [21]	0.871	0.934
Scope at mid-lumen time (%) (median [IQR])	12.5 [6]	11 [3]	12 [6]	0.551	0.687

The initial evaluation of Group A (ev-A) did show several significantly lower aptitudes among the novices compared to the moderately and highly experienced bronchoscopists. The identification score was significantly lower in ev-A compared to ev-B and ev-C (*p* = 0.001), as was the median overall performance score: 5.9% (IQR = 5.1) in ev-A compared to 25.3% (IQR = 13.8) in ev-B and 22.2%, IQR = 5.6) in ev-C (*p* = 0.0001). Total procedure time was also significantly longer for the novices, i.e. 561 s in ev-A vs 198 s in ev-B and 210 s in ev-C (*p* = 0.0001). In contrast, the visualization score, scope-wall contact time and scope at mid-lumen time did not differ significantly between the three groups for the initial evaluations (Table 2).

### Group a performance progression

**Fig. 2:**
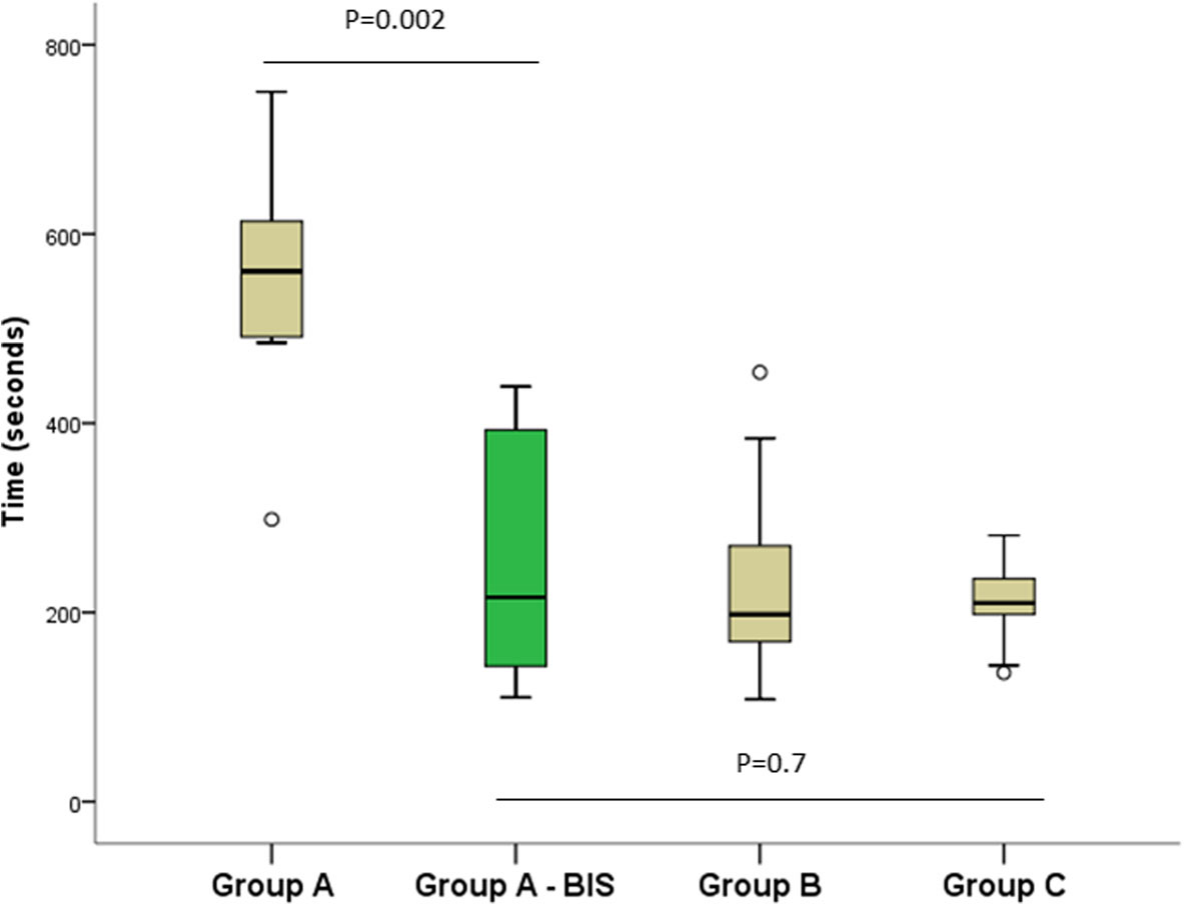
Distribution of total procedure times (in seconds) for groups A, B and C

The final evaluation of Group A (ev-A-bis), performed after the period of simulator self-training, showed significant improvements for most variables in comparison to the results of ev-A (Table 3 and Additional file 2: Figures S1-S3). The median total procedure time fell from 561 s (IQR = 134) in ev-A to 216 s (IQR = 257) in ev-A-bis (*p* = 0.002) (Fig. 2). The visualization and identification scores also improved significantly from ev-A to ev-A-bis. Resultantly, the overall performance score climbed from 5.9% (IQR = 5.1) in ev-A to 25.5% (IQR = 26.3) in ev-A-bis (*p* = 0.002), i.e. a progression of 435%.

**Table 3:** Comparison of performance of Group A in the initial and final evaluations

	Group A	Group A	*p* value
	Initial evaluation (*n* = 8)	Final evaluation (*n* = 8)	
Visualization score (median [IQR])	25 (6)	28 (0)	0.009
Identification score (median [IQR])	8 (11)	27 (4)	0.001
Total score (median [IQR])	33 (17)	55 (4)	0.001
Total procedure time in seconds (median [IQR])	561 (134)	216 (257)	0.002
Overall performance score (%) (median [IQR])	5.9 (5.10)	25.5 (26.3)	0.002
Scope at mid-lumen time (%) (median [IQR])	12.5 (6)	13.5 (4)	0.832
Scope-wall contact time (%) (median [IQR])	60.5 (18)	51.5 (9)	0.343

### Comparison of group A-bis (post simulator self-training) to groups B and C

Group A, after their simulator self-training (Group A-bis), correctly visualized significantly more anatomical structures (median 28 [IQR = 0] in ev-A-bis) than did Group B (median 27 [IQR = 1] in ev-B) (*p* = 0.017) and correctly identified significantly more structures (median 27 [IQR = 4] in ev-A-bis) than did Group C (median 20 [IQR = 9] in ev-C) (*p* = 0.02). There were no other statistically significant differences between Group A-bis and Groups B and C (Table 4). After the simulator self-training, the median overall performance scores also became comparable, i.e. 25.5% (IQR = 26.3) in ev-A-bis vs 25.3% (IQR = 13.8) in ev-B (*p* = 0.954) and vs 22.2% (IQR = 5.52) in ev-C (*p* = 0.564).

**Table 4:** Comparison of performance of Groups A-bis (post self-training, final evaluation), B and C (initial evaluations)

	Group A-bis	Group B	Group C	*P* value (A-bis vs B)	*P* value (A-bis vs C)
	No exp. ev. final (*n* = 8)	Moderate experience (*n* = 17)	Extensive experience (*n* = 9)		
Visualization score (median [IQR])	28 [0]	27 [1]	28 [3]	0.017	0.244
Identification score (median [IQR])	27 [4]	24 [8]	20 [9]	0.118	0.02
Total procedure time in seconds (median [IQR])	216 [257]	198 [112]	210 [77]	0.954	0.7
Overall performance score (%) (median [IQR])	25.5 [26.3]	25.3 [13.8]	22.2 [5.5]	0.954	0.564
Scope-wall contact time (%) (median [IQR])	51.5 [9]	62 [8]	63 [21]	0.07	0.247
Scope at mid-lumen time (%) (median [IQR])	13.5 [4]	11 [3]	12 [6]	0.208	0.498

### Satisfaction score

The median global satisfaction scores were 44 (IQR = 4) for Group A-bis, 38 (IQR = 8) for Group B, and 35 (IQR = 9) for Group C (*p* = 0.02) (maximum possible score = 48; Additional file 2: Figure S4). The results for the individual items in the satisfaction survey are provided in Additional file 2: Figure S5. The simulator’s contribution to future practice received a median score of 6 from Group A and 5 from Groups B and C (*p* = 0.009). Group A gave a median score of 5 for the simulator’s potential to reduce anxiety during future FB procedures; Groups B and C gave median scores of 4 and 2 for this item respectively (*p* = 0.009).

## Discussion

In the present study, we showed the interest of a FB self-training program employing a high-fidelity simulator: in one to three half-day training sessions, residents who were novices in bronchoscopy significantly improved their knowledge of endobronchial anatomy and their basic capacities for FB (visualization and identification of bronchial elements, procedure time). In our study, the basic capacities of the novices who followed the program became comparable to those of moderately (30–200 FBs) and even highly (> 200 FBs) experienced bronchoscopists.

The results of our work support those of Colt et al. [16], who tested a high-fidelity simulator FB training program with self-training*.* They too found significant post-training improvements in FB skills, including a reduction in the number of non-visualized segments (4.4 before training vs 0.8 after; *p* = 0.029). As also reported by Moorthy et al [26] and Colt et al [16], the performance scores of our novices after their training program (Group A-bis) became statistically comparable to those of our highly experienced bronchoscopists (Group C). .

In the initial evaluation, we could not discriminate skill levels using the visualization score. This is partially explained by the standardized examination conditions which do not entirely reflect the difficulties encountered in real FBs. We do however underline the statistically significant progression for the visualization score that we observed in Group A after the self-training. Thus, this aspect of performance constitutes a reachable learning objective for novice bronchoscopists.

The reduction in total procedure time observed after the simulation self-training is remarkable (median procedure time reduced by more than 50%). Such a reduction could result in more patient comfort [4] [5] and less complications [6] when newly trained bronchoscopists begin performing procedures in real patients.

The participants in all three groups expressed a high level of satisfaction with the simulation-based training process (Kirkpatrick level 1 evaluation [29]), suggesting good compliance for possible training programs to come. Similar observations were reported in the validation study for the Simbionix Bronch Mentor simulator [25].

One main limitation of all high-fidelity simulators is the cost of the equipment, maintenance and housing. Several ways can however limit this issue such as use low-fidelity simulators [15], or mutualize a high-fidelity simulator in a large area. In Lyon area, we chose to buy one simulator for 4 universities.

Out of the 22 initially invited novice bronchoscopists, only eight completed the self-training sessions and the final evaluation. Group A was thus limited to those eight participants. Several factors play a role in this loss of Group A participants but were unfortunately not fully assessed here. First, many of these latter were first-semester residents who could not find sufficient time to self-train on the simulator due to their hospital activities. Also, a certain number of those participants lived and/or practiced far away from the simulation center (> 2 h by car for some). In addition some participants experienced FBs on “true” patients after baseline evaluation and were thus not interested in following the full simulation-based training program; although local supervisors were fully implicated in this study and were aware to avoid proposing in-vivo FBs to these trainees.

Thus, continuing to subject patients to FBs performed by still-learning students seems undesirable in light of the development of simulation-based bronchoscopy training tools [24], the efficacy of which has been demonstrated in the literature [21, 30] and further reinforced with our study. For these ethical reasons, we chose to not include a demonstration of the transfer of simulation-acquired competencies to real practice, as this would require a control group of interventions by learners on real patients. However, Ost et al. did do that in their 2001 study [17].

The absence of a statistically significant difference for median performance between Groups B and C in our study does not necessarily indicate that our moderately experienced bronchoscopists have FB skills comparable to our highly experienced bronchoscopists. Indeed our study evaluated only endobronchial anatomical visualization and identification, crucial, but nonetheless relatively basic FB steps. We did not evaluate competencies for more complex procedures, such as biopsies or the extraction of foreign objects. We note particularly that the standardized nature of the examination conditions (normal bronchial tree, no coughing or bleeding) did not allow us to test participant competencies in emergency or stressful situations. However, an earlier work showed that the technical skills of experienced surgeons remain stable under stress whereas those of less experienced surgeons diminish [31]. It is thus probable that more experienced practitioners do better than their less-experienced counterparts for the managerial (team, procedure and task management) aspects when faced with urgent and/or stressful FB situations (hemoptysis, acute respiratory distress, etc.). These competencies, which were not evaluated in our work, are obviously essential in critical situations [32] and, when mastered, contribute greatly to the expertise of the practitioner.

## Conclusion

In conclusion, we showed here that freely accessible self-training on a high-fidelity bronchoscopy simulator enables the acquisition of basic flexible bronchoscopy skills needed for complete endobronchial exploration. In this study, novice bronchoscopists who trained themselves on a simulator acquired performance levels for rapidity, dexterity and precision similar to those of moderately and or even highly experienced bronchoscopists. Thus, the results of our study, combined with those of other studies, lead us to suggest that classical bronchoscopy training be replaced rapidly and obligatory by high-fidelity simulation-based training programs upstream of any interventions on real patients. The availability and accessibility of such programs would be greatly facilitated by mobile simulators.

## Additional files

Additional file 1: **Table S1.** Sequential steps during a normal flexible bronchoscopy procedure and correspondent skills to acquire. (DOCX 17 kb)

Additional file 2: **Figure S1.** Distribution of identification scores for Groups A, B and C. **Figure S2.** Distribution of visualization scores for Groups A, B and C. **Figure S3.** Distribution of overall performance scores (%) for Groups A, B and C. **Figure S4.** Distribution of global satisfaction scores for Groups A, B and C. **Figure S5.** Distributions for individual satisfaction survey items (*n* = 8, scored 0 (ineffective) to 6 (excellent)) for Groups A, B and C. **Figure S6.** Satisfaction survey. (DOCX 237 kb)

## Abbreviations

ERS: European Respiratory Society
FB: Flexible Bronchoscopy
HERMES: Harmonising Education in Respiratory Medicine for European Specialists
HFBS: High-Fidelity Bronchoscopy Simulator
