# Supplementary material for: High-fidelity simulation self-training enables novice bronchoscopists to acquire basic bronchoscopy skills comparable to their moderately and highly experienced counterparts
Source: BMC Med Educ. 2018 Aug 7;18:191. doi: 10.1186/s12909-018-1304-1 (PMC6081833; doi:10.1186/s12909-018-1304-1)
Supplement: Supplementary file 1 — Table S1. Sequential steps during a normal flexible bronchoscopy procedure and correspondent skills to acquire. (DOCX 17 kb) [file 12909_2018_1304_MOESM1_ESM.docx]

**Additional file 1**

| **Bronchoscopy steps** | **Knowledge assessed** | **Skill(s) assessed** |
| --- | --- | --- |
| Use of bronchoscope | - | Manipulate endoscope and its different functions (rotation, aspiration, up-down change of direction…) |
| Go through nasal cavity | Know normal anatomy of naso-pharyngeal cavities. | Demonstrate ability to find the correct way from nostril to vocal cords through naso-pharyngeal cavities. |
| Go through vocal cords | - | Demonstrate ability to go through vocal cords without causing injury / excessive cough. |
| Go down in the trachea | - | Demonstrate ability to safely navigate in bronchus/trachea with bronchoscope. |
| Explore the full right bronchial tree | Know normal right bronchial tree anatomy (sub-lobar level). | Demonstrate comprehensive right bronchial tree exploration, and accurate identification of lobar and sub-lobar bronchi |
| Go back to carena | - | Demonstrate ability to safely navigate in bronchus/trachea with bronchoscope. |
| Explore the full left bronchial tree | Know normal left bronchial tree anatomy (sub-lobar level). | Demonstrate comprehensive right bronchial tree exploration, and accurate identification of lobar and sub-lobar bronchi |
| Go back into trachea and vocal cords | - | Demonstrate ability to safely navigate in bronchus/trachea with bronchoscope. |
| Check vocal cords mobility | Know how checking vocal cords mobility. | Demonstrate ability to check vocal cords mobility. |
| Go back through nasal cavity | - | Demonstrate ability to safely navigate in bronchus/trachea with bronchoscope. |
| Local aneasthesia | Not assessed here | Not assessed here |
| Bronchus diseases recognition | Not assessed here | Not assessed here |
| Endobronchial exams (BAL, biopsy…) | Not assessed here | Not assessed here |

*Table S1 - Sequential steps during a normal flexible bronchoscopy procedure and correspondent skills to acquire.*
