# Supplementary material for: High-fidelity simulation self-training enables novice bronchoscopists to acquire basic bronchoscopy skills comparable to their moderately and highly experienced counterparts
Source: BMC Med Educ. 2018 Aug 7;18:191. doi: 10.1186/s12909-018-1304-1 (PMC6081833; doi:10.1186/s12909-018-1304-1)
Supplement: Supplementary file 2 — Figure S1. Distribution of identification scores for Groups A, B and C. Figure S2. Distribution of visualization scores for Groups A, B and C. Figure S3. Distribution of overall performance scores (%) for Groups A, B and C. Figure S4. Distribution of global satisfaction scores for Groups A, B and C. Figure S5. Distributions for individual satisfaction survey items (n = 8, scored 0 (ineffective) to 6 (excellent)) for Groups A, B and C. Figure S6. Satisfaction survey. (DOCX 237 kb) [file 12909_2018_1304_MOESM2_ESM.docx]

**Additional file 2**

**Figure S1: Distribution of identification scores for Groups A, B and C**

**Figure S2: Distribution of visualization scores for Groups A, B and C**

**Figure S3: Distribution of overall performance scores (%) for Groups A, B and C**

**Figure S4: Distribution of global satisfaction scores for Groups A, B and C**

**Figure S5: Distributions for individual satisfaction survey items (n=8, scored 0 (ineffective) to 6 (excellent)) for Groups A, B and C**

**Figure S6: Satisfaction survey**

*
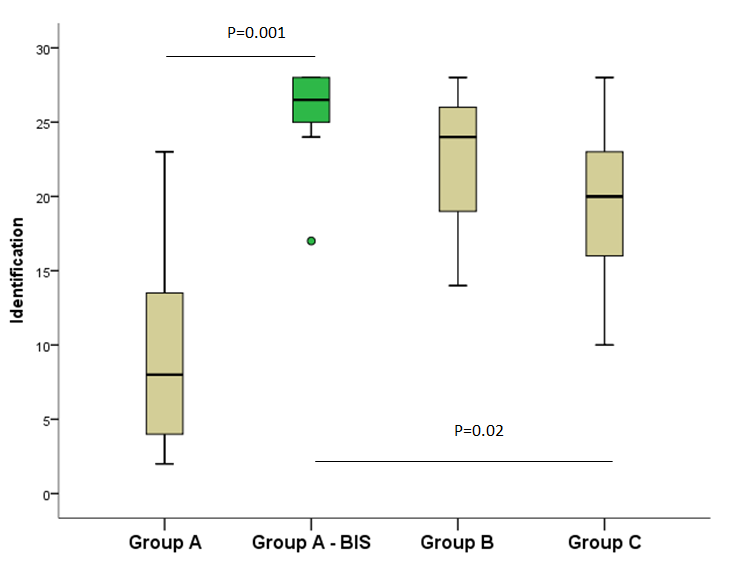
*

*Figure S1: Distribution of* ***identification scores*** *for Groups A, B and C*


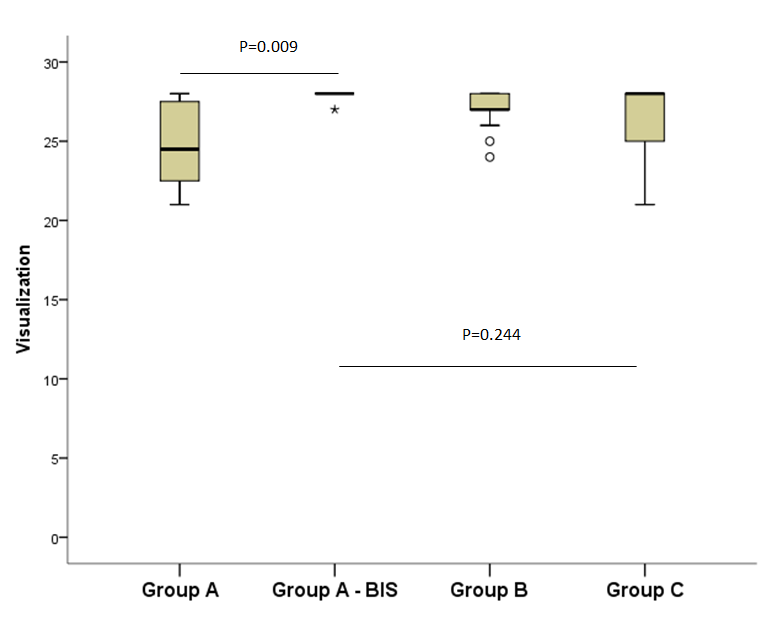


*Figure S2: Distribution of* ***visualization scores*** *for Groups A, B and C*

*
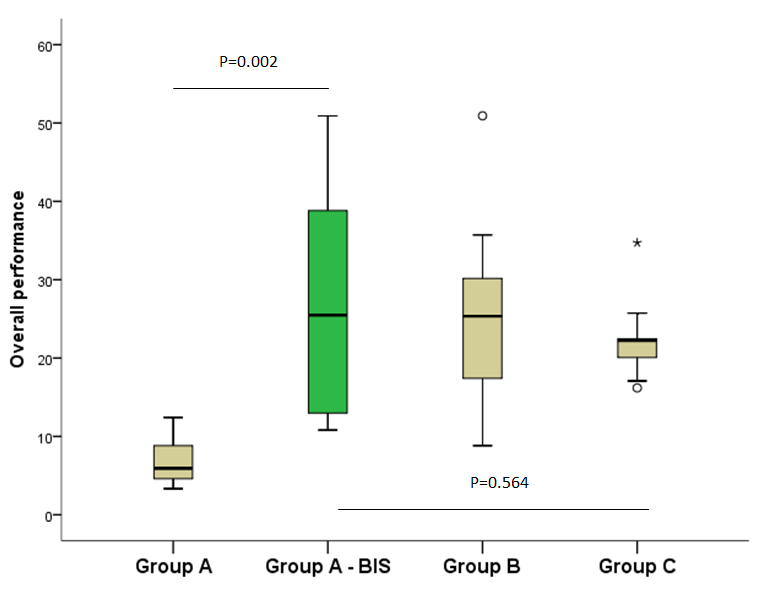
*

*Figure S3: Distribution of* ***overall performance scores*** *(%) for Groups A, B and C*


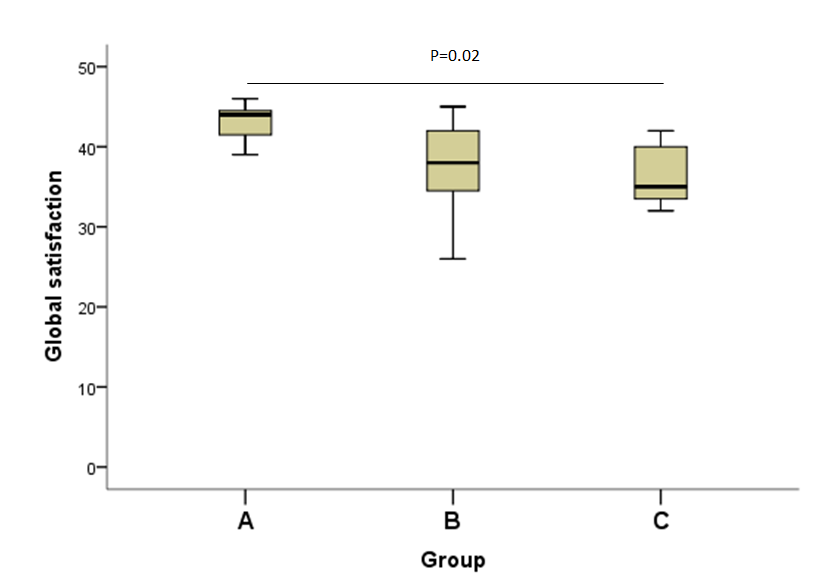


*Figure S4: Distribution of* ***global satisfaction scores*** *for Groups A, B and C*


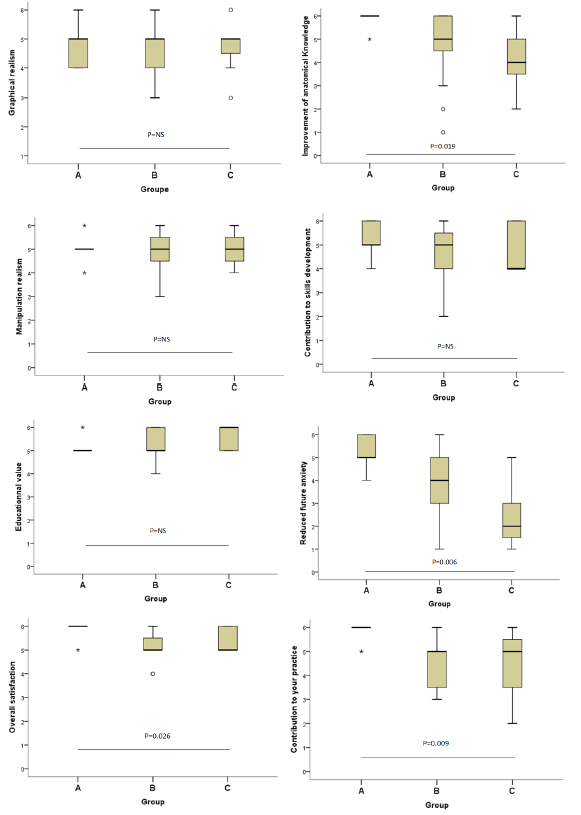
*Figure S5: Distributions for individual satisfaction survey items (n=8) scored 0 (ineffective) to 6 (excellent)) for Groups A, B and C*

Degree of overall satisfaction of the training

Contribution of the simulator to reduce your apprehension during your next endoscopic gestures

2

1

5

1

2

6

5

4

3

1

2

6

5

4

3

1

2

6

5

4

3

6

5

4

3

4

2

6

4

3

1

2

6

5

3

1

1

1

2

6

5

4

3

2

6

5

4

3

Contribution of the simulator for your future practice in endoscopy

Realism of the manipulation of the endoscope

Contribution on the improvement of your technical skills (precision, dexterity ...)

Quality of the pedagogical interface

Contribution on the improvement of your anatomical knowledge

Quality of the graphic realism

Each axis corresponds to an evaluation theme and an assessment of 0 to 6:

- 0 means Very bad

- 1 " Not satisfying

- 2 " Unsatisfying

- 3 " Satisfying enough

- 4 " Satisfying

- 5 " Very satisfying

- 6 " Excellent

*Figure S6 : Satisfaction survey*
